# Structure-informed evolutionary analysis of the meiotic recombination machinery

**DOI:** 10.1101/2025.11.07.687317

**Published:** 2025-11-10

**Authors:** Meret Arter, Jeffrey Vedanayagam, Min Lu, Momar Diop, Kaixian Liu, Eric C. Lai, Scott Keeney

**Affiliations:** 1 Molecular Biology Program, Memorial Sloan Kettering Cancer Center, New York, NY 10065; 2 Developmental Biology Program, Memorial Sloan Kettering Cancer Center, New York, NY 10065; 3 Howard Hughes Medical Institute, Memorial Sloan Kettering Cancer Center, New York, NY 10065

## Abstract

Despite being essential for fertility, many proteins involved in meiotic homologous recombination have diverged rapidly. The evolutionary forces driving this divergence remain mostly unknown, in part because of challenges in accounting for the interplay of sequence changes with constraints imposed by proteins’ structures and physiological roles. Here, we explore strategies to more sensitively detect signatures of positive or relaxed selection by integrating evolutionary analyses with structural and functional information, using meiotic recombination proteins in four taxa—primates, rodents, birds and budding yeasts. By mapping selection rate estimates onto predicted protein structures, we characterized protein regions likely to have experienced positive selection. We further identified subtle sequence variation within protein domains that are well conserved generally because of structural constraints. To detect sequence variation masked by these constraints, we analyzed selection at structurally matched residues, comparing homologs across different lineages as well as between meiosis-specific and generalist paralogs. These approaches identified lineage- and paralog-restricted enrichment of non-synonymous substitutions that may indicate loss of functional constraints and/or adaptive innovation. Finally, we used cross-species complementation experiments in *Saccharomyces cerevisiae* to show that sequence variation in the pro-crossover factor MSH4 modulates recombination proficiency. We suggest that evolutionary plasticity per se is a key conserved characteristic of the meiotic recombination machinery. More generally, our approach provides a mechanistic framework to analyze protein evolution.

## Introduction

To keep genome content stable across generations, sexually reproducing eukaryotes generate gametes that contain only half the parent’s chromosome number. Most organisms generate gametes through meiosis, a specialized cell division in which maternal and paternal homologous chromosomes usually use homologous recombination to pair, exchange genetic information, and then subsequently segregate (Sonntag Brown et al, 2013; Hunter, 2015).

**Fig. 1:**
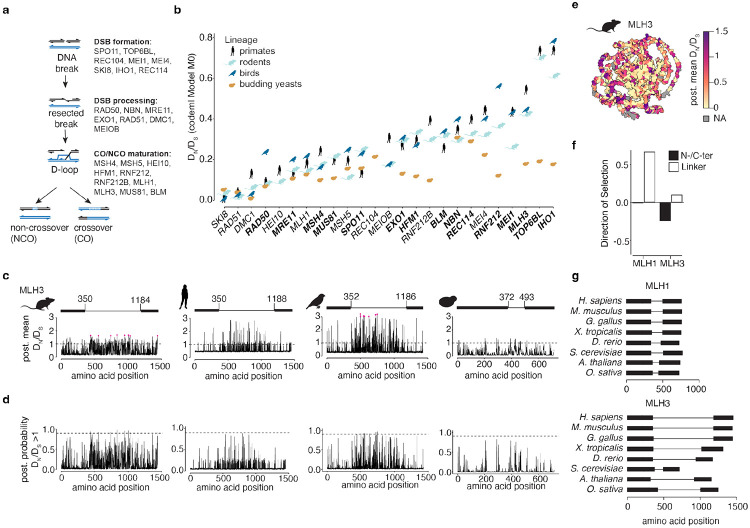
Structure-informed evolutionary analyses of the meiotic recombination machinery. **a)** Conserved DNA events and proteins required for meiotic recombination **b)** Average $D_{N}∕D_{S}$ ratio for each gene. The genes are ordered according to the average across the four lineages. Genes in bold showed significant signs of positive selection in at least one lineage (LRT for codeml site models M7 vs. M8). **c,d)** BEB estimation of post. mean $D_{N}∕D_{S}$ values **(c)** and post. probability for positive selection **(d)** for each residue in MLH3 in the four lineages. Residues with a post. probability > 0.9 for positive selection are highlighted in pink in **(c)**. Dashed lines indicate $D_{N}∕D_{S}=1$ **(c)** and post. probability = 0.9 **(d)**. The schematic above each graph in **(c)** shows positions of the N- and C-terminal folded domains (thick lines, from Interpro (Paysan-Lafosse et al, 2023)) and the central, unstructured linker (thin lines). **e)** Post. mean $D_{N}∕D_{S}$ mapped onto the AlphaFold model of mouse MLH3. The color scheme for mean $D_{N}∕D_{S}$ values is capped at 1.5. **f)** Direction of selection analysis in house mouse population data for the folded domains and unstructured linker regions of MLH1 and MLH3. **g)** Greater variation between organisms in length of the unstructured linker in MLH3 than in MLH1.

Meiotic recombination is initiated by DNA double-strand breaks (DSBs) generated by topoisomerase-like SPO11 and its partners (Lam & Keeney, 2014) (Fig. 1a). DSBs are resected by endo- and exonucleases (Ceccaldi & Cejka, 2025). The resulting long single-stranded 3’ DNA overhangs are bound by ssDNA binding proteins and RAD51-like recombinases that mediate invasion of the homologous chromosome to form branched recombination intermediates including D-loops, single-end-invasions, and double Holliday junctions (Brown & Bishop, 2014). Two main pathways result in either noncrossover or crossover outcomes. Noncrossovers form mostly through synthesis-dependent strand annealing, involving the unwinding of early recombination intermediates by DNA helicases (Allers & Lichten, 2001; McMahill et al, 2007). Crossovers are mostly formed through the asymmetric resolution of double Holliday junctions by nucleases (Schwacha & Kleckner, 1995). Only crossovers contribute to physical linkages between homologous chromosomes that are needed for segregation, but all recombination events contribute to chromosome pairing in many species, and all DSBs need to be faithfully repaired to maintain genomic integrity in all species.

Meiotic recombination is an ancient process that is shared across eukaryotes and has been studied in many organisms. Meiotic recombination errors are generally associated with missegregation of chromosomes, which can lead to aneuploid gametes and infertility (Hassold & Hunt, 2021). While the process overall is widely conserved, functional and genomic studies repeatedly revealed substantial variability within the protein machinery that performs each step (Arter & Keeney, 2024). Given that this machinery is important for the generation of viable gametes in the organisms studied, it is reasonable to assume that it was likewise important in their ancestors. Yet many of these proteins are poorly conserved, showing recurrent loss of individual proteins or protein families, emergence of lineage-specific proteins, changes in the meiotic function of conserved proteins, and/or evolutionarily rapid changes in amino acid sequences (Arter & Keeney, 2024).

It remains unclear how and why the meiotic recombination machinery is so evolutionarily plastic while continuing to maintain functionality over millions of years. An important step in answering this question is to understand at what rate and strength natural selection (purifying, neutral, or positive) is driving the evolution of these proteins. Using phylogenetic approaches, previous studies found signs of positive selection in several meiotic recombination proteins in different lineages such as flies, mammals or plants (Anderson et al, 2009; Sidhu et al, 2017; Brand et al, 2018; Dapper & Payseur, 2019; Szasz-Green et al, 2024; Zakerzade et al, 2025). However, most of these studies examined only a subset of the meiotic recombination machinery and they did not address which protein domains are most rapidly evolving.

Current strategies for detecting positive selection tend to work best for residues where recurrent positive selection leads to multiple amino acid substitutions; these strategies are often underpowered to detect other signatures of adaptive selection because of key limitations (Hughes, 2007). First, selective pressures will generally not be all-purifying or all-positive: even amino acid residues under positive selection are also likely to have constraints on how freely they can change while maintaining the protein’s function. Second, single codon analysis of selection has limited statistical power, but increasing the power by averaging over multiple residues or entire proteins comes at the cost of potentially mixing residues with different combinations of selection (positive, purifying, neutral). Third, treating proteins as strings of amino acids without regard to three-dimensional structure or biochemical function can obscure evolutionary signatures. Finally, experimental studies often sidestep evolutionary variability entirely by focusing on the most conserved proteins or protein domains.

To address these issues, we explored strategies for increasing the sensitivity for detecting signatures of positive or relaxed selection by combining systematic evolutionary genetic analysis with structural and other biochemical information about the target proteins’ functions. We focused on core components of the meiotic recombination machinery required for DSB formation, end resection, strand invasion, and processing of recombination intermediates into crossover and noncrossover products. Our results yield evidence for otherwise cryptic positive selection in multiple phylogenetic lineages and suggest hypotheses about which protein domains and molecular functions have been the focus of evolutionary change.

## Results

### Overview of approach

The codeml package of Phylogenetic Analysis using Maximum Likelihood (PAML) (Yang, 1997) estimates synonymous ($D_{S}$) and non-synonymous ($D_{N}$) substitution rates. Under the simplifying assumption that synonymous substitutions are unseen by selection, a relative enrichment of non-synonymous substitutions (higher $D_{N}∕D_{S}$ ratio) is a signature of reduced purifying selection often interpreted as positive selection. Codeml is a codon-based analysis that can be combined with protein structural models to generate further insights into which protein domains show signs of positive selection (Stern et al, 2007; Busset et al, 2011). The advent of high-quality structure prediction algorithms (Jumper et al, 2021; Varadi et al, 2022) greatly expands the ability to integrate PAML with structural information. A recent study used this approach to infer functionally relevant sites under positive selection in centromere proteins (Dudka et al, 2023).

Here we analyze meiotic recombination proteins in four eukaryotic lineages: primates, rodents, birds, and *Saccharomyces* budding yeasts (supplementary Fig. 1a-d). We integrated empirical or AlphaFold-generated structural models with PAML to determine how signatures of selection are shaped by structural constraints (folded domains vs. intrinsically disordered regions; buried/structural residues vs. protein-protein interfaces vs. solvent-exposed surfaces). We also sought to further disentangle positive selection signatures from the obscuring effects of purifying selection attributable to biochemical and structural constraints by comparing homologous proteins between different lineages and comparing paralogous protein pairs within lineages, where one paralog is (mostly) meiosis specific.

We selected 25 proteins that are directly involved in meiotic recombination (Fig. 1a,b) and mined Ensembl, NCBI Refseq, and UniProt databases and used blastn searches of published genome assemblies to identify high-confidence gene sequences. In total, we compiled 1156 sequences encoding 25 proteins from 58 species in 4 lineages (supplementary Table 1, supplementary Fig. 1a-d). For each lineage, we used MUSCLE (Edgar, 2004) to generate amino acid-based multiple sequence alignments that were then manually curated (see Methods).

### Many meiotic recombination proteins show signatures of positive selection and/or relaxed selection

We first ran codeml Model 0 to estimate an average $D_{N}∕D_{S}$ ratio for the entire coding sequence of each protein in each lineage dataset using species trees (Fig. 1b). Model 0 is a baseline model that estimates one $D_{N}∕D_{S}$ ratio averaged across all sites and all species in the phylogeny. Genes with the highest overall $D_{N}∕D_{S}$ were *IHO1*, *TOP6BL*, *MLH3,* and *RNF212*, while *SKI8*, *RAD51*, and *DMC1* showed the lowest ratio, reflecting high sequence conservation.

To examine selection within different domains, we next ran codeml site models, which allow the $D_{N}∕D_{S}$ ratio to vary across the gene and therefore allow detection of regions that are under positive selection in a gene that is overall under purifying selection. Site models treat the $D_{N}∕D_{S}$ ratio for any codon in the gene as a random variable from a statistical distribution and different models use different underlying statistical distributions for $D_{N}∕D_{S}$. Two pairs of nested models are most frequently used to test for sites under positive selection: models M1 (also called nearly neutral model) and M2 (positive selection), and models M7 (beta) and M8 (beta&ω). Only M2 and M8 allow for sites under positive selection. Likelihood ratio tests (LRTs) can evaluate which model better explains observed sequence variation. PAML and similar algorithms have been widely used to detect signatures of positive selection (Yang, 2002).

PAML statistics of all LRTs are summarized in supplementary table S2. Fifteen of the proteins analyzed had codons with significant evidence of positive selection in at least one lineage (testing M1 vs. M2 and M7 vs. M8): SPO11, TOP6BL, IHO1, REC114, MEI1, MRE11, RAD50, NBN, EXO1, MSH4, HFM1, RNF212, BLM, MUS81, and MLH3 (Fig. 1b). The rodent, bird, and primate datasets had 15, 7, and 6 proteins under positive selection, respectively, but no proteins scored as significant in yeast. Several of these proteins were identified in earlier studies in other lineages: RAD50 and MRE11 homologs in *Drosophila* (Anderson et al., 2009); MLH1, MRE11B, MUS81-1, and SPO11-2 in maize (Sidhu et al., 2017); and IHO1, MSH4, MRE11, and NBS1 in mammals (Dapper & Payseur, 2019). There was overall some, but not perfect, consistency in the ranking of the proteins according to the average $D_{N}∕D_{S}$ ratio even when signatures of positive selection did not rise to the level of significance in all lineages (Fig. 1b).

### Signatures of positive selection are enriched in a disordered region of MLH3

MLH3 showed significant evidence of positive selection in all three vertebrate lineages (P < 0.05 for M8 vs. M7; supplementary table S2), so we focused on this protein to explore the relationship of its domain architecture to its selection signatures. MLH3 is required for cross-over-specific resolution of recombination precursors (Zakharyevich et al, 2012; Cannavo et al, 2020) and loss of MLH3 leads to infertility in mice (Lipkin et al, 2002). The protein consists of a structured N-terminal domain containing an ATPase head and a transducer domain, as well as a structured C-terminal domain that is required for dimerization with MLH1. The two structured domains are connected by a long intrinsically disordered linker domain (Fig. 1c).

To see where residues that are predicted to be evolving under positive selection are located, we used posterior mean $D_{N}∕D_{S}$ ratios (from here on: post. mean $D_{N}∕D_{S}$) and the posterior probabilities that $D_{N}∕D_{S}$ is greater than 1 generated under Model 8 with Bayes empirical Bayes (Fig. 1c-d). Amino acid residues with a posterior probability >0.9 (post. probability, highlighted in pink in Fig. 1c), i.e., those that have the strongest support for being under positive selection, are all within the intrinsically disordered region of the protein. This region is also under less purifying selection in all lineages, as judged by higher post. mean $D_{N}∕D_{S}$ estimates, although no individual residues reached significance in primates or yeasts (Fig. 1c-d).

We therefore tested whether estimating $D_{N}∕D_{S}$ ratios separately for the structured and intrinsically disordered regions improves detection of selection signatures, using codeml fixed-site models (supplementary table S3). In this analysis, the linker region is under significantly less purifying selection than the structured domains in all lineages, even in yeast where there is not overall a significant signature of positive selection. MLH1, which shares the same domain structure, showed a similar pattern of less purifying selection in its linker domain (supplementary table S3). Integrating the post. mean $D_{N}∕D_{S}$ estimates with Alphafold structural models (in which the linker appears as a disordered ribbon orbiting the structured core) allows direct visualization of these connections between selection rates and domain organization (Fig. 1e).

One potential issue with disordered regions could be poor alignment quality, resulting in erroneously elevated $D_{N}∕D_{S}$ ratios. To mitigate this concern, we examined MLH3 and MLH1 sequence variation within a single species using mouse population genomic data (Harr et al, 2016). Since these sequences are close to identical, alignment errors are unlikely. We used DNAsp (Rozas et al, 2017) to analyze intra- and interspecies variation in the linker region and the structured N- and C-terminal domains. In both MLH1 and MLH3, the linker shows more intraspecies variation (Fig. 1f), suggesting that the enrichment of linker sequence divergence within lineages is not (solely) a consequence of alignment ambiguity. In addition to sequence variation, the MLH3 linker also shows length variation between widely diverged species (Furman et al, 2021) (Fig. 1g). Interestingly, a large part of the linker in mouse (and other species) is encoded by one very long first exon.

Overall, MLH3 provides an instructive example of significant evidence for recurrent positive selection particularly enriched in an intrinsically disordered region. Similar enrichment in intrinsically disordered regions was observed for other proteins in our dataset, including pro-crossover factor HFM1 or the non-crossover-promoting helicase BLM (supplementary fig. 2a-d).

### Evaluating sequence divergence against a backdrop of structural constraints

**Fig. 2:**
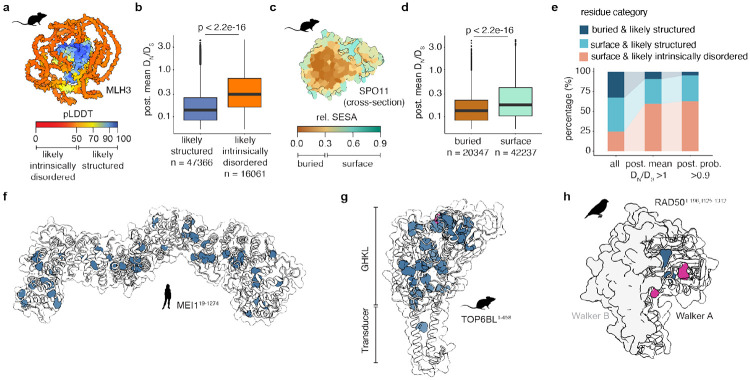
Structural constraints influence selection rates. **a)** AlphaFold model of mouse MLH3 (from Fig. 1e) colored by prediction confidence score pLD-DT. **b)** Post. mean $D_{N}∕D_{S}$ values for residues categorized by pLDDT as likely structured or likely intrinsically disordered. The p value is from a t test. Here and throughout, the horizontal lines in boxplots indicate median and interquartile range, whiskers indicate 1.5 times the interquartile range, and outliers are shown as individual points. **c)** A cross-section of mouse SPO11 AlphaFold structure (AF-Q9WTK8-F1), colored by the relative solvent exposed surface area (rel. SESA). **d)** Post. mean $D_{N}∕D_{S}$ values for residues categorized by rel. SESA as buried or surface. **e)** Percentages of structured or intrinsically disordered residues in the complete dataset (all residue, all proteins, all lineages) or among only the residues that show relaxed purifying selection (post. mean $D_{N}∕D_{S}>1$) or positive selection (post. probability > 0.9). **f-h)** Transparent surface and ribbon representations of Alphafold models of human MEI1 **(f)**, mouse TOP6BL **(g)**, and zebra finch RAD50 **(h)**. Residues that show relaxation from purifying selection (post. mean $D_{N}∕D_{S}>1$) despite strong structural constraints (buried & likely structured) are in blue (shown as spheres for better visibility, residues R3 and S53); residues in pink (RAD50 M94 and A149) are also statistically significant (post. probability > 0.9).

Disordered regions are, by definition, subject to low structural constraint, allowing many of their residues to vary without negatively impacting protein function. Thus, much if not all of the variation in the disordered regions of MLH3, HFM1, and BLM could be due simply to less purifying selection because of less structural constraint (Brown et al, 2011; Afanasyeva et al, 2018). To explore this possibility more widely, we asked how the AlphaFold “predicted local distance difference test” score (pLDDT) relates to $D_{N}∕D_{S}$ for each residue. A low pLDDT (< 50) can be used as a predictor of disorder (Jumper et al., 2021) (Fig. 2a). When grouped according to this threshold, residues in presumptively intrinsically disordered regions indeed showed higher $D_{N}∕D_{S}$ estimates on average across all genes. This is true across all lineages and within each lineage considered individually (Fig. 2b and supplementary Fig. 2e).

By similar reasoning, buried residues are expected to experience stronger structural constraints than surface residues (Franzosa & Xia, 2009; Echave et al, 2016). As a measure for an amino acid’s location, we used the relative solvent exposed surface area (rel. SESA) for each residue (Methods) (Fig. 2c). When residues were grouped into buried (rel. SESA < 0.3) and surface categories (rel. SESA >= 0.3), surface residues showed higher $D_{N}∕D_{S}$ estimates on average, as expected (Fig. 2d and supplementary Fig. 2f).

In the complete dataset, 29.3% of residues were classified as both buried and likely structured (Fig. 2e). By contrast, residues in this category were a smaller fraction of those residues that showed evidence of relaxed selection (8.5% for those with post. mean $D_{N}∕D_{S}>1$) or that more stringently showed evidence of positive selection (4.6% for those with post. probability > 0.9) (Fig. 2e). Conversely, residues classified as surface-exposed and likely intrinsically disordered were overrepresented among residues showing evidence of relaxed or positive selection. Despite this global tendency, we categorized substantial numbers of residues as both buried and likely structured, but that also showed signatures consistent with either relaxed or positive selection (e.g., n = 280 residues with post. mean $D_{N}∕D_{S}>1$; n = 9 residues with a post. probability > 0.9). We consider here several illustrative examples.

The proteins with the most such residues are MEI1 and TOP6BL, which were also among the fastest evolving proteins in our dataset. Although its specific biochemical function remains unclear, MEI1 is required for meiotic DSB formation in mice and *A. thaliana* (Libby et al, 2003; Vrielynck et al, 2021) and mutations in human *MEI1* are associated with infertility (Zhang et al, 2023). Nearly all of MEI1 is predicted to be structured, containing Armadillo-like folds, but 46 residues with a post. mean $D_{N}∕D_{S}>1$ are distributed throughout the core of the structure (Fig. 2f).

TOP6BL is a direct binding partner of SPO11 related to the GHKL-family ATPase subunit of archaeal topoisomerase VI (Robert et al, 2016; Vrielynck et al., 2021). When present, TOP6BL orthologs are essential for DSB formation in every organism tested (Arter & Keeney, 2023). It has multiple residues that are buried and structured and that also show evidence of positive selection (n = 35 in the rodent dataset and n = 19 in the primate dataset). In rodents, most of these residues are in and around the degenerate GHKL (1-172) as well as at the top of the transducer domain (196-450) (Fig. 2g). These regions overall show more sequence variation than the lower part of the transducer domain, which mediates the interaction with SPO11 (supplementary Fig. 2g). This variation is interesting because the GHKL domain has been lost independently in several lineages (*Drosophila*, *Schizosaccharomyces, Saccharomyces*) (Brinkmeier et al, 2022; Yu et al, 2024).

Remarkably, of the nine residues in the full dataset that scored as buried and structured but also showed significant evidence of positive selection (post. probability >0.9), four are in RAD50 in the bird lineage. RAD50 homologs are universally present in eukaryotes, prokaryotes, and archaea. In eukaryotes, RAD50 is a component of the MRX/N complex and has roles in the early DNA damage response, DSB repair, DSB end resection, and (in some organisms) meiotic DSB formation (Mimitou & Symington, 2009; Hopfner, 2023). Two of those four residues (M94 and A149) in RAD50 locate to the N-terminal ATPase domain (Walker A motif), while the other two residues (A332, R788) are located in the central flexible coiled coil domain (Fig. 2h, supplementary Fig. 2h). The Walker A domain also contains two additional residues (R3 and S53) that are classified as buried and likely structured and show post. mean $D_{N}∕D_{S}>1$ without reaching significance. Components of the MRX/N complex were previously flagged to be under positive selection in mammals and *Drosophila* (Anderson et al., 2009; Dapper & Payseur, 2019), but it was unclear which protein domains were involved.

### Structural and functional constraints in protein-protein interaction surfaces

**Fig. 3:**
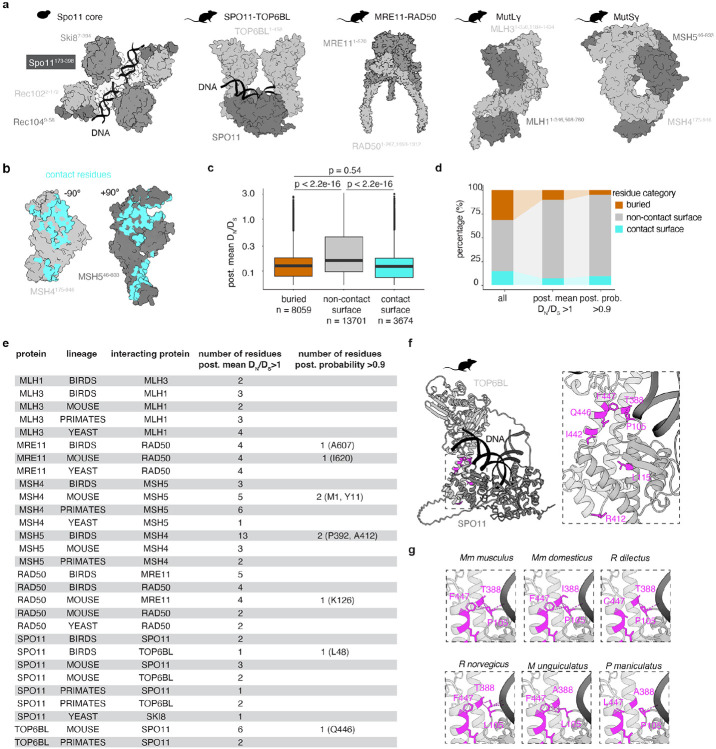
Purifying selection associated with protein-protein interaction surfaces. **a)** Examples of protein complex structures from cryo-EM or AlphaFold3 prediction. Disordered regions are omitted for clarity. **b)** MSH4-MSH5 contact residues (cyan) in mouse MutS**γ**. The proteins are shown as in **(a)**, but each subunit is rotated 90° left or right to reveal the interaction surfaces. **c)** Post. mean $D_{N}∕D_{S}$ values for residues characterized as either buried, noncontact surface, or contact surface. The p value is from a t test. **d)** Percentages of buried, noncontact surface, or surface residues in the complete dataset or among only the residues that show relaxed purifying selection (post. mean $D_{N}∕D_{S}>1$) or positive selection (post. probability > 0.9). **e)** Table summarizing the contact-surface residues with post. mean $D_{N}∕D_{S}>1$ and post. probability > 0.9. **f)** Overview (left) and detailed view (right) of the interaction between mouse SPO11 and TOP6BL. Only one dimer of SPO11–TOP6BL is shown for simplicity. Interfacial residues with post. mean $D_{N}∕D_{S}>1$ are highlighted in magenta. **g)** Predicted interaction of SPO11-P105 with TOP6BL-T388 and F447 in *M. musculus musculus* (pink dashed lines, Van der Waals overlap ≥ −0.4 Å). Six distinct amino acid combinations in these three residues are present in the rodent lineage. Four combinations are predicted to partially or completely abolish this interaction when using the swapaa command in ChimeraX. SPO11-P105 is also predicted to interact with the DNA backbone (shown in black).

We also evaluated sequence variation within the context of constraints imposed by protein-protein contacts. Similar to structured and buried regions, protein surfaces that mediate protein-protein interactions were found to experience increased purifying selection (Guharoy & Chakrabarti, 2010). Our dataset contains four multiprotein complexes: dimers of the SPO11 core complex (the core complex comprises SPO11-TOP6BL heterodimers in vertebrates, or heterotetramers of Spo11, Rec102, Rec104, and Ski8 in yeast), the MRX/N complex (MRE11 and RAD50 plus NBN or yeast Xrs2), MutSɣ (MSH4-MSH5), and MutLɣ (MLH1-MLH3). We generated models for each complex using AlphaFold3 (Abramson et al, 2024) (Fig. 3a) then identified interfacial contact residues, defined as those that are solvent accessible for a protein in isolation but buried within the complex (Fig. 3b) (Meng et al, 2023). On average across all complexes in the full dataset, contact residues show significantly lower $D_{N}∕D_{S}$ than other surface residues. In fact, their average is comparable to constitutively buried residues (Fig. 3c).

Thus, as expected, direct protein-protein contacts provide additional structural constraints that we can leverage to give context for interpreting sequence variation. Despite these constraints, and as with constitutively buried residues described in the preceding section, we again identified multiple contact residues displaying indices of relaxed purifying selection (n = 96 residues with post. mean $D_{N}∕D_{S}>1$), out of which 9 residues showed significant signs of positive selection (n = 9 with post. probability > 0.9). The nine significant residues were found in interacting regions of MRE11, RAD50, TOP6BL, MSH4, and MSH5 in different lineages (Fig. 3d-e, supplementary table S4).

We consider here the mouse SPO11 core complex as an illustrative example. We identified two interfacial residues in mouse SPO11 (P105, L115) with post. mean $D_{N}∕D_{S}>1$, and six in TOP6BL (T388, F447, Q446, I442, R412). Four of the TOP6BL residues (T388, I442, Q446, and F447) cluster in the center of the transducer domain in a region that interacts with loops in the winged-helix domain of SPO11 (Fig. 3f). Interestingly, two of these residues (TOP6BL-T388 and -F447) are predicted to interact directly with one of the variable SPO11 residues (P105) (Fig. 3g). Furthermore, the amino acid combinations present in several other rodent species are predicted to alter or disrupt the interaction (Fig. 3g). A fifth variable TOP6BL residue (R412) and the second SPO11 residue (L115) do not contact one another directly, but both are part of a common contact surface comprising an alpha-helical bundle that is critical for the SPO11-TOP6BL interaction (Fig. 3f) (Humphreys et al, 2021; Brinkmeier et al., 2022; Yu et al., 2024; Zheng et al, 2025). The SPO11-SPO11 dimeric interface also showed three residues with post. mean $D_{N}∕D_{S}>1$ (T195, S197 and L375) (Supplementary Fig S3a). For the same interface in other lineages, primate TOP6BL T388 is conserved as A387, F447 is conserved as F446, Q446 is conserved as Q445, I442 is conserved as I441, and R412 is conserved as R411. For SPO11, P105 and L115 are conserved within primates and birds as well. Within primates two other residues in the vicinity in SPO11 (Q109, S112) show post. mean $D_{N}∕D_{S}>1$ and are also in contact with TOP6BL. TOP6BL itself is conserved at the SPO11 contact surface.

### Leveraging paralogous proteins to highlight otherwise cryptic sequence variation

Proteins that have different biochemical roles may experience different mixes of selection pressures. For example, proteins (or domains within proteins) that have enzymatic activity may be more constrained on average than proteins that serve solely as structural binding partners. Because the variable backdrop of purifying selection may obscure signs of positive selection, we reasoned that it would be advantageous to compare paralogous proteins with similar structures and biochemical properties, but that function in different cellular contexts.

**Fig. 4:**
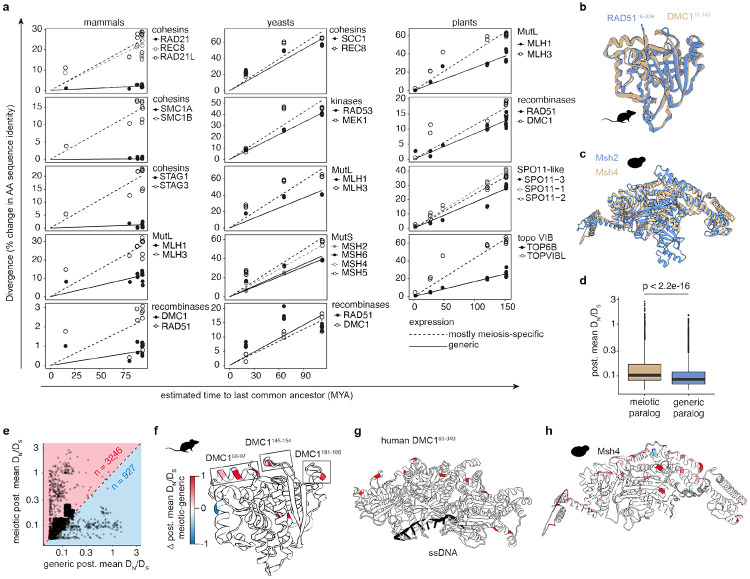
Paralog comparisons reveal subtle sequence divergence. **a)** Pairwise divergence for paralogous protein pairs. Linear regression lines generated in R. Solid circles/lines: generic expression, open circles/dashed lines: mostly meiosis-specific expression. Analyzed species are *Saccharomyces arboricola*, *S. kudriavzevii*, *S. cerevisiae*, *S. eubayanus*, *Nauvomozyma castellii*, and *Kluyveromyces lactis* for yeasts, *Arabidopsis thaliana*, *Brassica napus*, *B. rapa*, *Nicotiana tabacum*, *Oryza sativa* and *Zea mays*, for plants, and *Homo sapiens*, *Mus musculus*, *Rattus norvegicus*, *Bos taurus*, and *Canis lupus* for mammals. **b-c)** Pairwise structural alignment of *M. musculus* RAD51 (blue) DMC1 (brown) in **(b)** and budding yeast Msh2 (blue) and Msh4 (brown) **(c). d)** Post. mean $D_{N}∕D_{S}$ values for all residues in the dataset that could be structurally matched between paralogous proteins. The p value is from a t test. **e)** Scatter plot showing post. mean $D_{N}∕D_{S}$ values for the same data as in **(d)**. For more residues, the post. mean $D_{N}∕D_{S}$ is higher in the meiotic paralog (n=3246), but 927 residues have higher post. mean $D_{N}∕D_{S}$ in the generic paralog. **f)** Mouse DMC1, colored by the difference (Δ) in post. mean $D_{N}∕D_{S}$ between structurally matched residues. Red color indicates higher post. mean $D_{N}∕D_{S}$ in DMC1 while blue color indicates more variability in RAD51. Three loops that show sequence variation specifically in DMC1 are highlighted. **g)** The residues of interest identified in **(f)** highlighted in red on a crystal structure of human DMC1 bound to single stranded DNA (PDB: 7C9C). **h)** Yeast Msh4, colored as in **(f)** based on comparison with Msh2.

A previous study in mammals found that paralogs that are largely or exclusively meiosis-specific tend to diverge faster (measured as the change in amino acid identity vs. time from last common ancestor) than their counterparts (referred to below as “generic” for simplicity) that have functions outside of meiosis (Boekhout et al, 2019). We extended these analyses to additional paralog pairs in plants and budding yeast, finding the same conclusion to apply in nearly all cases (Fig. 4a). Given the broad reproducibility of this trend, we incorporated paralog comparisons into our analyses.

In a first step, we tested if the strictly meiotic proteins DMC1 (meiosis-specific strand exchange protein) and MSH4 (a subunit of the MutSγ complex ) show higher $D_{N}∕D_{S}$ ratios than their generic paralogs RAD51 and MSH2 (subunit of MutSα and MutSβ (Groothuizen & Sixma, 2016), respectively. As predicted, codeml fixed-site models improve when we allow for different $D_{N}∕D_{S}$ ratios between the two genes in each meiotic vs. generic paralog pair in all lineages except for RAD51 vs. DMC1 in the bird lineage (supplementary table S5). In addition, the meiotic paralog shows a higher average $D_{N}∕D_{S}$ value in all cases except yeast Rad51 and Dmc1 (supplementary table S5).

Armed with this information, we then set out to directly compare structurally matched residues between paralogs, reasoning that such a comparison would help control for the differences in selection pressures experienced by different types of proteins and by residues residing in different biochemical/functional contexts. To do this, we generated pairwise structure alignments for RAD51 vs. DMC1 (Fig. 4b) and MSH2 vs. MSH4 (Fig. 4c), yielding 4267 residues that could be matched with high confidence between the paralogs in the four lineages. In agreement with the gene-level models, the residues of the meiotic paralogs show a significantly higher post. mean $D_{N}∕D_{S}$ on average (Fig. 4d). When $D_{N}∕D_{S}$ values for individual pairs of structurally matched residues are examined, 3246 show higher $D_{N}∕D_{S}$ in the meiotic paralog and only 927 are higher in the generic paralog (Fig. 4e).

We further mapped the differences between meiotic and generic $D_{N}∕D_{S}$ values onto the Alphafold structures (Fig. 4f-h). This analysis highlighted a set of rapidly diverging residues located near one another on a surface of rodent DMC1 (Fig. 4f). Closer inspection showed that one residue (R94), shows post. mean $D_{N}∕D_{S}>1$, and two more (T148 and E182) are just below that threshold ($D_{N}∕D_{S}=0.943$ and 0.891 respectively) (Supplementary Fig. S4a-b). In contrast, the equivalent residues are widely conserved in DMC1 in the other lineages. This surface is opposite the ssDNA-binding region, such that these variable residues spiral around the nucleoprotein filament formed by cooperative assembly of DMC1 on ssDNA (Fig. 4g).

As a second example we analyzed yeast Msh4 (Fig. 4h). MSH4 showed significant signs of positive selection in the primate and rodent lineages but not in yeast (supplementary Fig. S4c-d, supplementary Table S2). Msh4 contains many residues that are specifically under less purifying selection in Msh4 than in Msh2, but unlike in mouse DMC1, they are more broadly distributed throughout the structure (Fig. 4h, Supplementary Fig. S4e-f).

### Functional impact of sequence variation in budding yeast MSH4

**Fig. 5:**
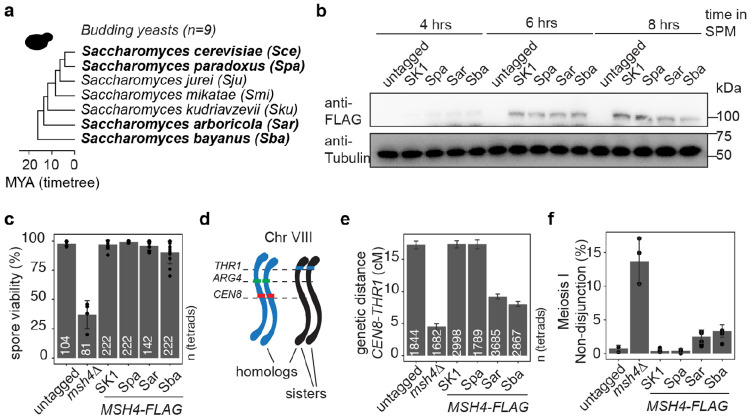
Cross-complementation assays with budding yeast Msh4. **a)** Endogenous *MSH4* in *S. cerevisiae* was replaced with FLAG-tagged versions of *MSH4* from diverged *Saccharomyces* species (bold). **b)** Immunoblot analysis of Msh4-FLAG expression at the indicated times during meiosis. **c)** Spore viability. Bars show the pooled means, error bars indicate SDs and points indicate individual experiments. The total number of tetrads dissected is indicated for each genotype. **d)** Spore-autonomous fluorescent constructs allow the scoring of crossing over in the *CEN8–THR1* interval. **e)** Genetic distance from *CEN8* to *THR1*. Bars indicate genetic distances and error bars are standard errors. The total number of tetrads for each genotype is indicated. At least two independent experiments were performed per genotype. **f)** Meiosis I non-disjunction was calculated from the same tetrad data as in **(e)**. Bars show the pooled means, error bars indicate SDs and points indicate individual experiments.

To test whether the sequence variation in yeast Msh4 affects function, we performed cross-complementation experiments using FLAG-tagged *MSH4* gene sequences from *Saccharomyces* species *S. paradoxus*, *S. bayanus* and *S. arboricola* expressed in *S. cerevisiae* under the control of the endogenous *MSH4* promoter (Fig. 5a). Immunoblots confirmed expression and stability of the proteins in synchronously sporulating cultures (Fig. 5b). For each strain, we assessed spore viability and crossing over within the *CEN8:THR1* genetic interval on chromosome VIII using spore-autonomous expression of fluorescent proteins (Thacker et al, 2011) (Fig. 5c-e-f).

*MSH4* from *S. cerevisiae* and *S. paradoxus* supported wild-type spore viability and crossing over. The diverged *MSH4* sequences from *S. arboricola* and *S. bayanus* complemented most of the defect seen with a *msh4* null mutation, indicating that both species’ proteins retain much of their function in *S. cerevisiae*. However, both yielded a small but significant decrease in both viability and crossing over, with the defect in spore viability correlating with an increased frequency of meiosis I non-disjunction (Fig. 5c,e-f). We conclude that at least some of the sequence divergence in MSH4 is functionally relevant in the yeast lineage.

### Lineage specificity of rapid sequence change

Previous studies found that signs of positive selection were often restricted to specific lineages (Anderson et al., 2009; Sidhu et al., 2017; Dapper & Payseur, 2019; Szasz-Green et al., 2024). Our whole-gene evolutionary analysis similarly showed different levels of purifying, relaxed, or positive selection between lineages for several proteins (Fig. 1c), and the sequence variation discussed above for the SPO11–TOP6BL interface (Fig. 3f) and loop elements in DMC1 (Fig. 4f) is specific for rodents. We examine here several additional examples.

**Fig. 6:**
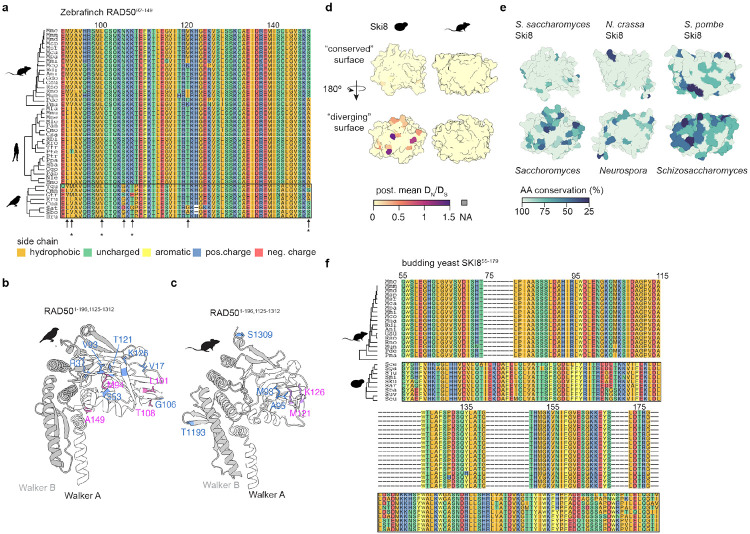
Lineage specificity of sequence variation. **a)** Partial alignment of the Walker A domain of RAD50. Amino acids are colored according to biochemical side chain properties (Hydrophobic: I, A, V, L, P, G, M; uncharged: Q, T, C, N, S; aromatic: Y, F, W; positive charge: R, H, K; negative charge: D, E). Black rectangle indicates the lineage in which we initially observed relaxation from purifying selection. Arrows indicate residues with post. mean $D_{N}∕D_{S}>1$ and asterisks indicate residues that also have post. probability > 0.9. **b,c)** RAD50 ATPase domains of zebra finch **(b)** and mouse **(c)**. Blue residues show post. mean $D_{N}∕D_{S}>1$; and pink residues also show post. probability > 0.9. **d)** Post. mean $D_{N}∕D_{S}$ mapped onto the Alphafold model of budding yeast Ski8 (left) and mouse WDR61 (right). Surface representations are matched between the species: N-terminal half above (more conserved in yeast), C-terminal half below (more diverged in yeast). **e)** Alphafold models of *Neurospora crassa* Ski8 (AF-Q7RVN5-F1) and *S. pombe* Ski8 (AF-Q09150-F1) together with *S. cerevisiae* Ski8 from **(d)**. Structures are colored by percent amino acid identity based on multiple sequence alignments (*N. crassa, N. cerealis, N. tetrasperma, N. Africana, N. sublineolata,* and *Sordaria macrospora* for the *Neurospora model*; *S. pombe, S. japonicus, S. cryophilus,* and *S. octosporus* for the *Schizosaccharomyces* model. **f)** Partial alignment of the N-terminal half of yeast Ski8 with equivalent region of rodent WDR61 (the primate and bird sequences are nearly identical to the rodent sequences).

As described above, the N-terminal ATPase domain of RAD50 in birds has two buried residues with post. mean $D_{N}∕D_{S}>1$ (zebrafinch R3 and S53) and two residues with post. probability > 0.9 (M94 and A149; Fig. 2h). In addition, seven surface residues in this domain (V17, S53, V93, L101, G106, T108, T121) also have post. mean $D_{N}∕D_{S}>1$ in birds, with post. probability > 0.9 for residues 101 and 108 (Fig. 6a,b). This domain also displays substantial variability in rodents but affecting mostly different residues: mouse M93 and A95 have post. mean $D_{N}∕D_{S}>1$ and M121 and K126 additionally have post. probability > 0.9 (Fig. 6a,c). Additionally, we identified two residues (T1193, S1309) in the mouse Walker B motif with $D_{N}∕D_{S}>1$. By contrast, the whole domain is highly conserved in both primate and yeast lineages (post. mean $D_{N}∕D_{S}<1$ for all residues; Fig. 6a and Supplementary Fig. 5a).

Ski8 in budding yeast provides another example of lineage-specific sequence variation. Ski8 directly interacts with Spo11 and is essential for meiotic DSB induction (Arora et al, 2004). This role is conserved in *S. pombe* and *Sordaria macrospora* (Evans et al, 1997; Tesse et al, 2003). However, Ski8 is dispensable for meiosis in *A. thaliana* (Jolivet et al, 2006), and it is unclear whether its orthologs have meiotic roles in metazoans or other lineages. Another function of Ski8 is more widely conserved: it forms a complex with Ski2 and Ski3 that influences RNA metabolism by the exosome (Halbach et al, 2013). Ski8/WDR61 was one of the most conserved proteins in our dataset, showing strong purifying selection (Fig. 1c).

Nonetheless, Ski8 in the *Saccharomyces* clade displayed multiple variable residues, most of which were confined to one half of the toroidal structure formed by the protein’s WD40 repeats (Fig. 6d, supplementary Fig. 5b-c). The same surface is completely conserved in the rodent lineage (Fig. 6c). In addition to these residues on the protein surface, several buried residues in the same part of the protein showed evidence of relaxed purifying selection as well (post. mean $D_{N}∕D_{S}>0.4$; supplementary Fig. 5b-c). Overall, we found that despite strong purifying selection across the whole protein, the N-terminal half of Ski8 (*S. cerevisiae* residues 12-179) has significantly higher $D_{N}∕D_{S}$ when using PAML fixed-site models (supplementary Table S3). Notably, the variable half of Ski8 is surface-exposed in both the Spo11 core complex and the Ski2–Ski3–Sk8 complex, whereas the more conserved face interacts directly with Ski2 in the Ski complex (supplementary Fig. 5d-e). We also observed surface variability in this half of the protein in other fungal lineages (namely, *Neurospora* and *Schizosaccharomyces*; Fig. 6e). The budding yeast protein also has several insertions of loops between the highly structured beta-sheets (visible as gaps in the other species in the alignment) (Fig. 6f). By contrast, WDR61 is almost perfectly conserved across the whole set of mammalian species, indicating high levels of purifying selection (Fig. 6f).

## Discussion

In this study, we explored how sequence evolution correlates with measures of intrinsic disorder, buried versus surface amino acid side chains, and the interfaces of protein-protein contacts within complexes. As anticipated, residues displaying a greater degree of structural and biochemical constraints were more likely to also display stronger purifying selection, but there were also many exceptions to this general feature. Our results show the value in integrating structural information to contextualize protein sequence changes with respect to domain architecture and biochemical function, and to unmask signs of relaxed or positive selection that may be obscured against a backdrop of purifying selection. AlphaFold greatly expands the information available for this contextualization, and we anticipate that our approach can also be extended more broadly to other tools for evolutionary analysis beyond PAML. We further anticipate that the strategies explored here will be valuable discovery tools to document evolutionarily relevant sequence changes and to design targeted genetic experiments.

One promising strategy we employed was the direct comparison of paralogous proteins that are matched for structural and functional properties and that have been evolving within the same species (Boekhout et al., 2019). Such comparisons provide a controlled like-for-like match of residues that are in a similar structural and biochemical context and are in proteins that perform similar molecular tasks. The approach thus has the potential to highlight sequence variation that is functionally significant even if positive selection is difficult to detect with evolutionary analysis methods that have limited statistical power. This approach should be broadly applicable to any paralog pairs, and it may also extend to comparisons between homologous domains of non-paralogous proteins.

Many proteins lack paralogs within the same species. With these cases, an alternative is to systematically compare between lineages for matched residues of orthologs. As with paralogs, this approach also controls for the backdrop of functional constraints while searching for signatures of positive selection. The lineage-specific examples we document here (the SPO11–TOP6BL interface; loop elements in DMC1; the RAD50 ATPase domain; and a specific surface on Ski8) join results from earlier studies (Anderson et al., 2009; Sidhu et al., 2017; Dapper & Payseur, 2019; Szasz-Green et al., 2024). Because each lineage represents a distinct evolutionary trajectory with different histories of selection pressures and adaptations, lineage specificity per se may thus be a useful hallmark to infer the functional relevance of observed sequence variation, and to highlight candidates for mechanistic follow-up studies.

Our data add to growing evidence that the meiotic recombination machinery, despite its essential functions for fertility, has pervasively experienced rapid evolution including relaxed purifying selection and/or positive selection (reviewed in (Arter & Keeney, 2023)). For several proteins, their rapid evolution contributes directly to variation in the recombination process itself. For example, changes in the DNA-binding sequence specificity of the histone methyltransferase PRDM9 causes changes in the distribution of recombination events across the genomes of many vertebrates (Baudat et al, 2010; Grey et al, 2018), and variation in amino acid sequence and/or gene copy number of the SUMO E3 ligase RNF212 is associated with variation in global crossover frequencies (Kong et al, 2008; Kadri et al, 2016; Johnston et al, 2018).

In most cases, however, the causes and consequences of rapid evolutionary changes of meiotic recombination proteins are not known. The sequence divergence we observe could be a result of relaxed purifying selection, adaptation to endogenous or exogenous selection pressures, genetic drift, increased mutation rates, or a combination of those. Several specific selection pressures have been proposed to play a role in shaping the recombination machinery: karyotype changes, the evolution of sex chromosomes, genomic conflict or meiotic drivers, and environmental effects including temperature (discussed in (Arter & Keeney, 2023)). Interestingly, two of the examples highlighted here have antiviral functions in certain lineages in addition to meiotic recombination functions. Antiviral functions as seen in host-pathogen conflicts are another classic driver of rapid evolution. First, the MRN complex inhibits adenovirus infection in mammalian cells and is in turn targeted for downregulation by a virus-encoded factor (Stracker et al, 2002; Schwartz et al, 2007). Second, the Ski2–Ski3–Ski8 complex reduces copy number and suppresses expression of double-stranded RNA viruses in *S. cerevisiae* (thus the “superkiller” phenotype when Ski proteins are missing because of overexpression of a virus-encoded secreted toxin) (Wickner, 1996). It is thus interesting to consider that lineage-specific signatures of rapid evolution of these factors might reflect selection pressures related to host-pathogen conflicts, meiosis roles, or both.

## Materials and Methods

### Sequence curation

Protein coding sequences for genes of interest were collected from NCBI, Ensembl and UniProt databases (O’Leary et al, 2016; Yates et al, 2020; UniProt, 2021). We used the isoform that had been confirmed experimentally, or, in the absence of such information, the longest annotated isoform. A list of gene sequence and Alphafold structure identifiers is in supplementary table S1. Our initial database contained a list of 1415 protein coding sequences covering 91 genes from 4 lineages. Out of those, 240 sequences were re-annotated using blastn because they were either mis-annotated or not previously annotated. Overall, 111 sequences could not be identified, likely due to gaps in the genome assemblies. Additional sequences were excluded from further analyses if they contained large gaps, leading to a total of 259 missing sequences. Because the robustness of PAML parameter estimation is strongly dependent on sequence number we further excluded any gene from a lineage with less than five homologous sequences available. Codon-guided multiple DNA sequence alignments were generated using MUSCLE as implemented in the R muscle package (Edgar, 2004). Multiple sequence alignments were then manually curated to remove gaps and alignment errors. Sequence alignments are available on Figshare (https://figshare.com/projects/Structure-informed_evolutionary_analysis_of_the_meiotic_recombination_machinery/263293).

### Phylogenetic analysis using maximum likelihood

#### PAML site models.

Phylogenetic analyses using maximum likelihood were performed using codeml from the PAML 4.9 suite (Yang, 1997). Species trees (supplementary Fig. S1a-d) were generated with timetree (Kumar et al, 2022). To check whether the sequence divergence was comparable between lineages (and thus suitable for PAML analysis) we first ran pairwise codeml (model 0) to estimate pairwise synonymous and non-synonymous substitution rates (supplementary Fig. S1e) (Yang, 1997; Anisimova et al, 2001, 2002). We then used codeml models M1, M2, M7 and M8 for likelihood ratio tests (LRTs) and estimation of post. mean $D_{N}∕D_{S}$. Site models allow the $D_{N}∕D_{S}$ ratio to vary for sites across the gene. Models 1 (nearly neutral) and 2 (positive selection), 7 (beta) and 8 (beta&ω) are nested models designed to test for signatures of positive selection. Since only models 2 and 8 allow for sites under positive selection, we can use LRTs and the LRT statistic (twice the log-likelihood difference between the two compared models (2Δℓ)), and a chi-square test to see which model is favored. LRTs to detect signatures of positive selection were performed using codeml.

#### Structure-informed PAML.

For structure-informed analyses we used the approximate post. mean $D_{N}∕D_{S}$ ratio (ω) computed using the Bayes empirical Bayes (BEB) method under the codeml model M8 as an estimate of selection. We mapped these estimates as attributes onto the Alphafold protein structures using ChimeraX (Meng et al., 2023) and custom R scripts. All Alphafold protein models downloaded from the database were version 4. A similar approach has previously been implemented in the Selecton server (Stern et al., 2007). To make visual inspection more straightforward we use a fixed color scheme across all structures with mean $D_{N}∕D_{S}$ capped at 1.5. Since post. mean $D_{N}∕D_{S}$ estimates for single residues are based on few datapoints, their accuracy is limited and highly sensitive to the number of sequences, distance between sequences, and selection. To see how robust estimation is within our dataset, we compared post. mean $D_{N}∕D_{S}$ estimates generated under M8 using the empirical Bayes method with estimates generated under different codeml models (M1, M2, M7 and M8 using naïve empirical Bayes or BEB) (supplementary Fig. 1f). We used the post. mean $D_{N}∕D_{S}$ estimates generated under M8 for further analyses. For consistency with previous publications, residues with a post. probability higher than 0.9 that $D_{N}∕D_{S}$ is greater than 1 were considered significant. While empirical Bayesian methods suffer from inaccuracy in small datasets, we deem it useful to distinguish between strong purifying selection and loss of purifying selection, especially when combined with structural and functional information about the amino acid residues to judge how much purifying selection to expect based on the mechanistic/biochemical context. All custom R scripts required for the analyses are available on Figshare (https://figshare.com/projects/Structure-informed_evolutionary_analysis_of_the_meiotic_recombination_machinery/263293).

#### PAML fixed-site models.

To perform statistical tests comparing selection rates between paralogous proteins or between specified domains or sections of proteins, we used partition gene models using the PAML fixed-site models as previously described (Yang & Swanson, 2002). Protein domain boundaries were obtained from Interpro (Paysan-Lafosse et al, 2023).

### Analyses of the mouse population genomic data

Population genomic data for 23 mouse strains were downloaded as VCF files from https://wwwuser.gwdguser.de/~evolbio/evolgen/wild-mouse/vcf/GVCF/ (Harr et al., 2016). The population samples were from France (14, 15B, 16B, 18B), Iran (413, 416, AH15, AH23, JR11, JR15), Kazakhstan (AL16, AL19, AL1), Czech Republic (CR12, CR13, CR16, CR23, CR25, CR29), India (H12, H36), and Germany (TP121B, TP1). The variants were extracted from the VCF file for each strain and reconstituted to the mm10 reference genome using the *vcf_to_fasta.sh* script, which utilizes the GATK *FastaAlternateReferenceMaker* tool (McKenna et al., 2010). The mm10 RefSeq genes were then extracted from the variant reconstituted genomes using the bedtools *getfasta* tool (Quinlan and Hall, 2010). Next, we performed McDonald-Kreitman tests (McDonald and Kreitman, 1991) with the *MK.pl* script, using *M. spretus* (SP36 strain) as an outgroup. All custom scripts required for the analyses are available on Figshare. The direction of selection metric (Stoletski and Eyre-Walker, 2011) was then calculated using the following formula:

$$\mathrm{DoS}=\frac{D_{N}}{D_{N}+D_{S}}-\frac{P_{N}}{P_{N}+P_{S}},$$

where $D_{N}$ and $D_{S}$ are divergent non-synonymous and synonymous sites, respectively, and $P_{N}$ and $P_{S}$ are polymorphic non-synonymous and synonymous sites. We used DNAsp to estimate direction of selection (Rozas et al., 2017).

### Protein structure prediction and visualization

Protein structural models were downloaded from the Alphafold database (Varadi et al., 2022). Models were not available for *T. guttata* RAD50, DMC1, HFM1, BLM, RNF212B, MLH3 and MUS81, these proteins were modelled using the Colabfold workbooks and the UniProt amino acid sequence (Mirdita et al, 2022). In this case the first model was chosen for each protein. Protein structure visualization and analyses of solvent exposed surface areas (SESA) were performed in UCSF Chimera (Pettersen et al, 2004) or ChimeraX (Meng et al., 2023). We calculated a relative SESA using theoretical maximum values for each amino acid, as previously described (Ma & Wang, 2015)

For the yeast Spo11 core complex we used a dimer prediction based on the published cryo-EM structure (Yu et al., 2024). For other protein complexes, we used predictions generated by the Alphafold3 online server (https://alphafoldserver.com/) (Abramson et al., 2024). Interaction surfaces were identified using ChimeraX by selecting residues that bury ≥ 15 Å^2^ of solvent-accessible surface in the interface between chains. We then grouped all residues into three categories: buried (relSESA < 0.3), surface (relSESA ≥ 0.3 in the monomeric form), and contact surface (relSESA ≥ 0.3 in the monomeric form, with interactions in the complex structure). Across the four lineages and four complexes, we classified a total of 3674 contact surface residues. We used the ChimeraX swapaa function to analyze how the different amino acid combinations found in different rodent species affect these interactions.

For pairwise structure alignments we used the FATCAT server (https://fatcat.godziklab.org) with default parameters using the flexible alignment mode (Ye & Godzik, 2004). Only high-confidence alignments were included for further analyses (Tm-score>0.5 and sufficient coverage of the whole protein sequence). Our final dataset included comparisons between RAD51 and DMC1 as well as MSH2 and MSH4. Calculation of the difference in post. mean $D_{N}∕D_{S}$ between structurally matched residues was performed in R using custom scripts. All scripts and resources are available from Figshare (https://figshare.com/projects/Structure-informed_evolutionary_analysis_of_the_meiotic_recombination_machinery/263293).

### Cloning and strain construction

All strains were SK1 derivatives, as detailed in Supplementary Table S6. The following alleles have been published previously: spore-autonomous fluorescent markers *THR1::m*-*Cerulean*-*TRP1*, *CEN8::tdTomato*-*LEU2*, and ARG4::GFP*-*URA3* (Thacker et al., 2011). To replace the endogenous copy of *MSH4*, *MSH4* sequence was cloned into a plasmid containing a FLAG tag and HpHMX resistance cassette by PCR. Subsequent gene replacements were then performed using In-Fusion cloning (Takara). Linearized vectors were then transformed into the wild-type SK1 background.

### Protein analyses by immunoblot

Samples for protein analyses were prepared from synchronously sporulating yeast cultures. In brief, diploid colonies were grown on YP-glycerol plates (2% peptone, 1% yeast extract, 2% glycerol, 2% agar) for 48 hrs at 30°C. Cells were then transferred onto YPD plates (2% peptone, 1% yeast extract, 2% dextrose, 2% agar) and grown to form a small patch (overnight, 30°C). Cells were then transferred to YPD plates and grown into a lawn overnight, 30°C), which was then used to inoculate pre-sporulation medium YP2%KAc (2% peptone, 1% yeast extract, 2% potassium acetate) to OD_600_ ~0.3. Cells were grown for 11 hr at 30°C, washed with sporulation medium (SPM, 2% potassium acetate) and inoculated into SPM to OD_600_ ~3.5. This time point was defined as t = 0 in all meiotic experiments. Ten ml of culture was taken to prepare TCA protein extracts. Samples were disrupted using a bead beater and glass beads in 10% trichloroacetic acid. Precipitates were resuspended in 2× NuPAGE sample buffer and neutralized with 1 M Tris base. Samples were heated (95 °C) and cleared by centrifugation. Samples were loaded on NuPAGE 4–12 % Bis-Tris (Invitrogen). Gels were blotted on PVDF membranes using semi-dry transfer (GE Healthcare). HRP-conjugated anti-FLAG antibody (Sigma A8592) was used for detection of Msh4. Rat anti Tubulin Alpha (Biorad, MCA78G) antibody was used for loading controls. Blots were imaged on a BioRad ChemiDoc system.

### Analysis of recombination using spore-autonomous fluorescence

The spore-autonomous fluorescence recombination analysis was performed as described previously (Thacker et al., 2011). Yeast cells were synchronized as described above and inoculated into SPM to OD_600_ ~3.5. Cells were incubated at 30 °C for ~48 hrs and subsequently imaged on a Zeiss Axio Observer Z1 Marianas Workstation, equipped with an ORCA-Flash 4.0 camera, illuminated by an X-Cite 120 PC-Q light source, with 60× air objective. Marianas Slidebook (Intelligent Imaging Innovations) software was used for acquisition. The pattern of fluorescence in the tetrads was manually scored using Fiji. Only tetrads with four spores and each fluorescence marker occurring in two spores were included in the final analysis. Recombination frequency, expressed as map distance in centimorgans with standard error, was calculated as described (Thacker et al., 2011) and using the Stahl online tools (https://elizabethhousworth.com/StahlLabOnlineTools/compare2.php).

### Spore viability

To assess spore viability, diploid yeast strains were sporulated on SPM plates (2% potassium acetate) overnight at 30 °C. Tetrads were treated with Zymolyase 100T (Zymo research) for 10 min at room temperature to disrupt the cell wall. Spore viability was determined by microdissection using a Singer SporePlay+ Dissection instrument.

## Supplementary Material

Supplement 1

Supplement 2
