# Supplementary material for: Structure-informed evolutionary analysis of the meiotic recombination machinery
Source: bioRxiv. 2025 Nov 10:2025.11.07.687317. Preprint. [Version 1] doi: 10.1101/2025.11.07.687317 (PMC12642689; doi:10.1101/2025.11.07.687317)
Supplement: Supplement 2 [file media-2.pdf]

Supplemental Information for:

Structure-informed evolutionary analysis of the meiotic recombination machinery

Meret Arter, Jeffrey Vedanayagam, Min Lu, Momar Diop, Kaixian Liu, Eric C. Lai, and Scott Keeney

This pdf contains:

Supplemental Figures S1–5
Supplemental Table S6

Supplemental Data provided as a separate file:

Supplemental Tables S1-5

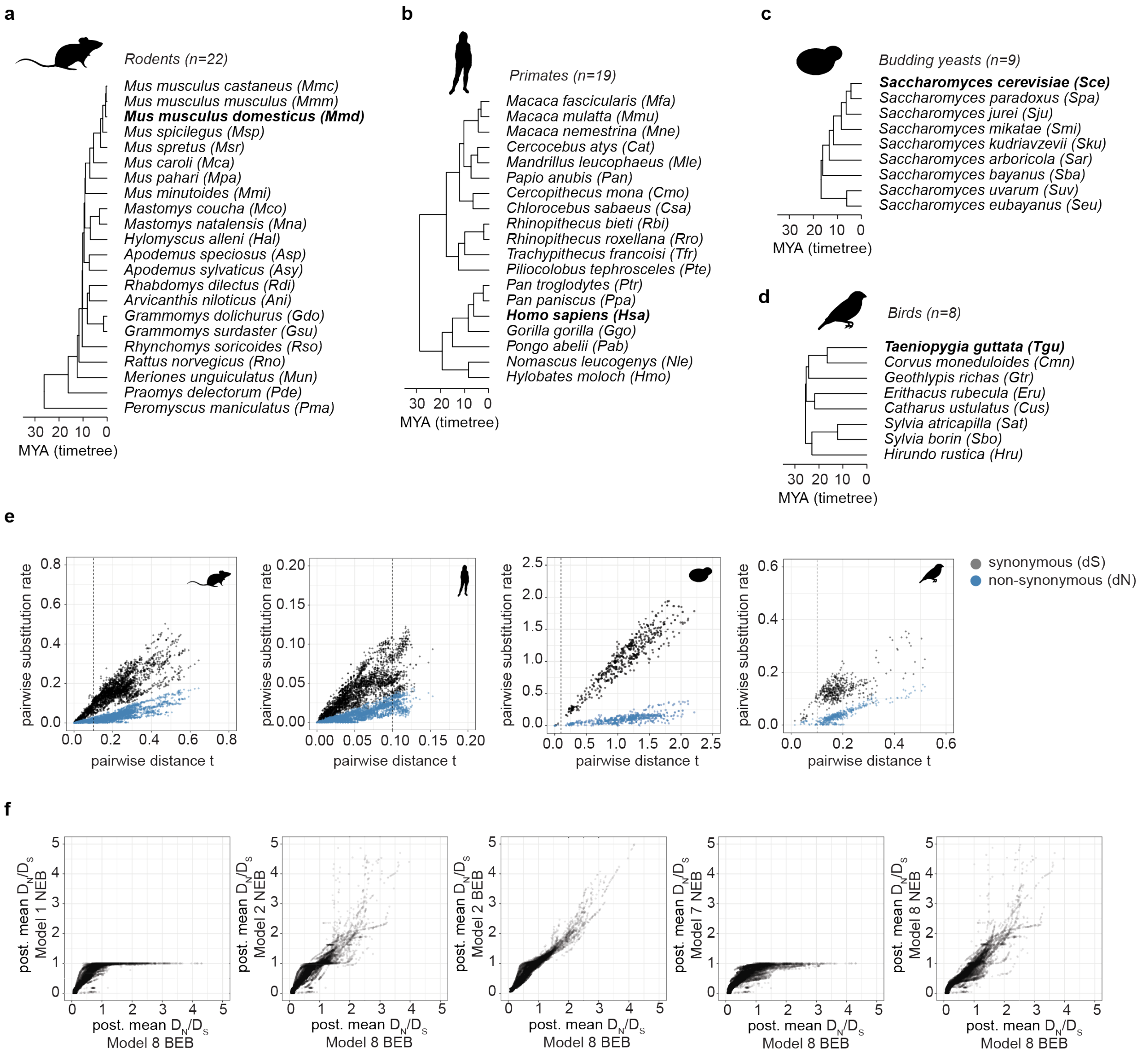

Fig. S1: Species trees and pairwise substitution rates. a-d) Species trees with bold text indicating representative species for which Alphafold models were obtained. Trees were generated using timetree (Kumar *et al*, 2022). MYA, million years ago. **e)** Pairwise synonymous (grey) and non-synonymous (blue) substitution rates plotted against the pairwise distance t for all genes. The vertical dashed lines in each plot indicate t = 0.1. **f)** Correlation of post. mean D_N/D_S estimates generated for each codon under different codeml models. Note that Models 1 and 7 do not allow for sites with D_N/D_S > 1.

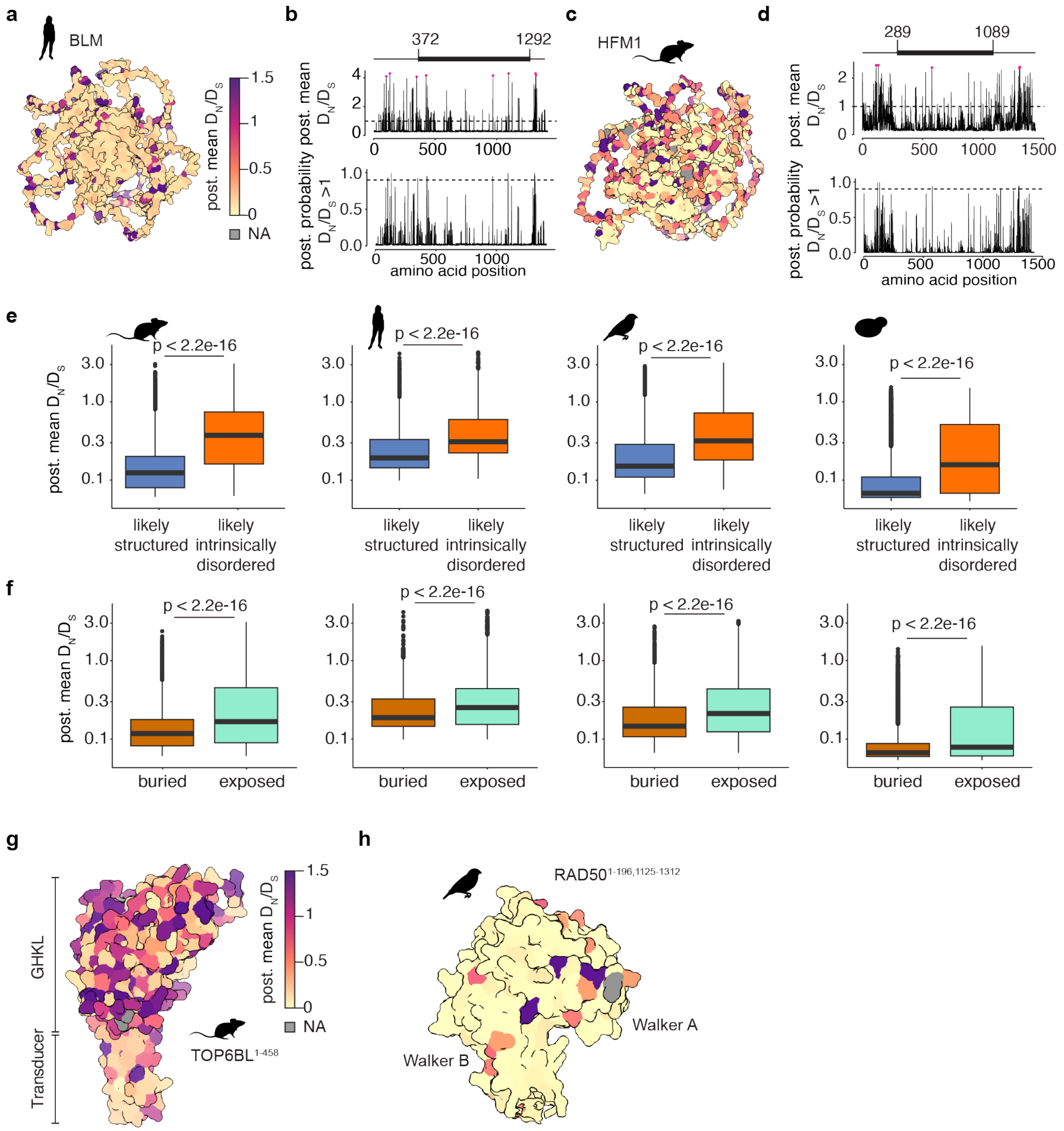

Fig. S2: Structural constraints influence selection rates. a-d) Analysis of primate BLM **(a,b)** and rodent HFM1 **(c,d)**. Structural models of human **(a)** or mouse **(c)** proteins are colored according to post. mean D_N/D_S. The graphs in (b) and (d) show post. mean D_N/D_S (top) and post. probability for positive selection (bottom) for each residue. Residues with a post. probability > 0.9 are shown in pink. Horizontal dashed lines indicate $D_N/D_S = 1$ or post. probability = 0.9. The schematics above in **(b,d)** show positions of the central structured domains (thick lines) and unstructured N- and C-terminal domains (thin lines). **e,f)** For each lineage, post. mean D_N/D_S values for residues categorized according to pLDDT as likely structured or likely intrinsically disordered **(e)** or categorized according to rel. SESA as buried or surface **(f)**. **g,h)** Post. mean D_N/D_S mapped onto AlphaFold models of mouse TOP6BL **(g)** and the zebra finch RAD50 head domain **(h)**.

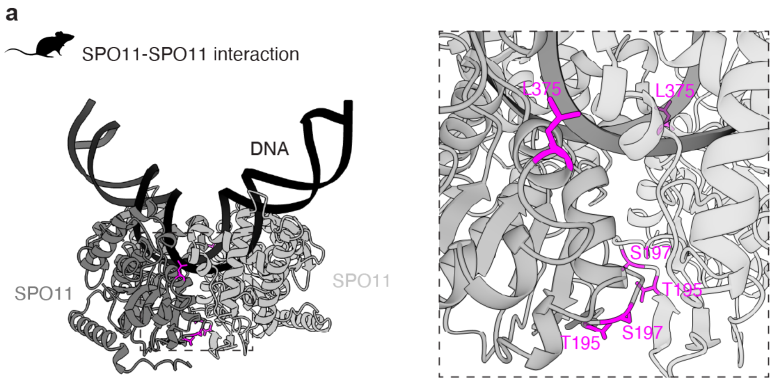

Fig. S3: Variation of the SPO11–SPO11 dimer interface in rodents. a) Overview and detailed view of the Alphafold structure of two SPO11–TOP6BL complexes bound to bent DNA. TOP6BL is omitted to highlight the SPO11 dimer configuration. Residues at the dimer interface that showed post. mean D_N/D_S > 1 (T195, S197 and L375) are highlighted in magenta.

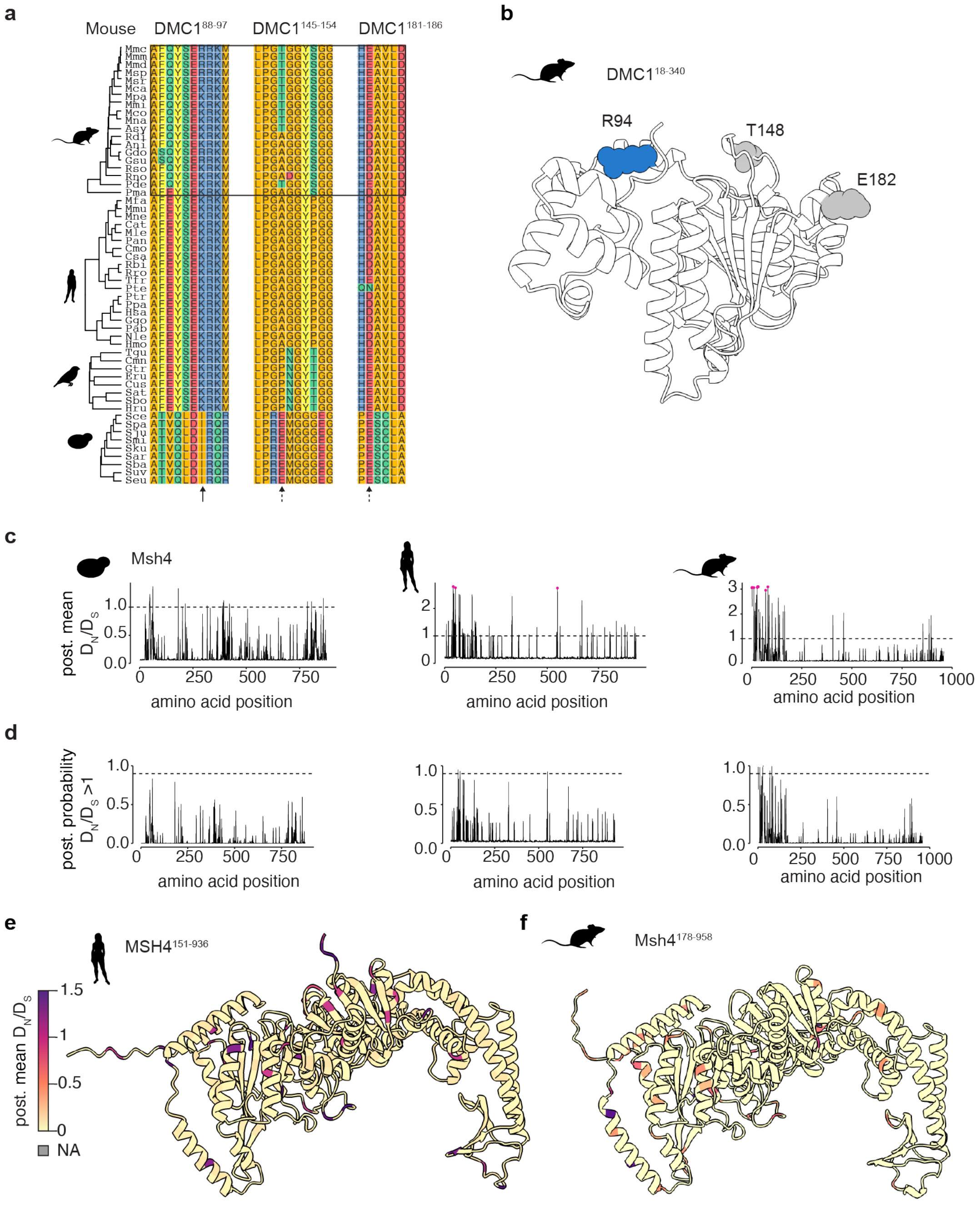

Fig. S4: Paralog comparisons. a) Partial alignment of DMC1. Black rectangle highlights the rodent lineage in which we initially observed relaxed purifying selection. Solid arrows indicate residues with post. mean D_N/D_S >1 and dashed arrows show residues with D_N/D_S just below 1. **b)** Mouse DMC1. The blue residue (R94) shows post. mean D_N/D_S > 1; gray residues have D_N/D_S just below 1 (T148, E182). **c,d)** Post. mean D_N/D_S **(c)** and post. probability for positive selection **(d)** for each residue in MSH4 in three lineages. Residues with a post. probability > 0.9 are shown in pink. Horizontal dashed lines indicate D_N/D_S = 1 or post. probability = 0.9. **e,f)** Post. mean D_N/D_S mapped onto the AlphaFold models of *H. sapiens* MSH4 **(e)** and *M. musculus* MSH4 **(f)**.

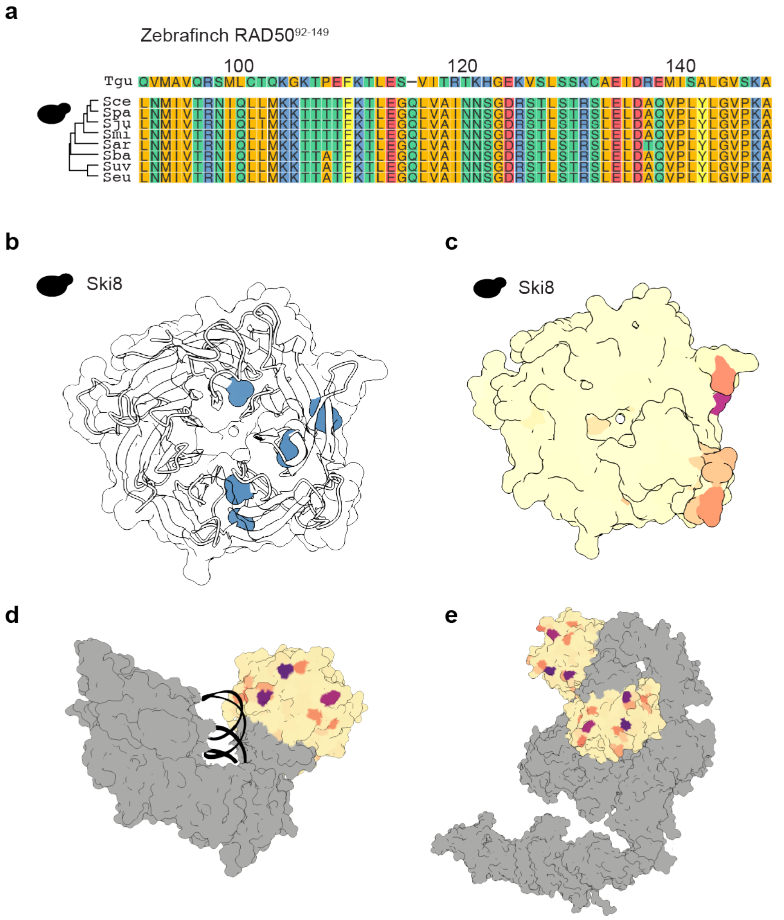

Fig. S5: Variation within Ski8 in budding yeasts. a) Partial sequence alignment of Ski8 in budding yeasts and Zebrafinch for comparison. **b)** Variation of structured residues. A transparent surface and ribbon representation of budding yeast Ski8 is shown. The five blue residues (H58, L81, V142, G198, V382) show a subtle relaxation from purifying selection (post. mean D_N/D_S > 0.4) despite strong structural constraints. **c)** Variation of surface residues. Post. mean D_N/D_S is mapped onto a surface representation of the AlphaFold model of budding yeast Ski8. **d,e)** Orientation of Ski8 in the context two complexes: the Spo11 core complex (monomeric cryo-EM structure from (Yu *et al*, 2024)) in **(d)** and the Ski2–Ski3–Ski8 complex (PDB: 4BUJ) in **(e)**. Only Ski8 is colored by post. mean D_N/D_S to highlight the position of the diverging surface.

Supplemental Table S6: List of yeast strains.

Strain	MAT	Genotype	Source/Ref.
SKY7454	a/α	wild type SK1 diploid	This study
SKY7421	a/α	MSH4(SK1)-FLAG-HpHMX4	This study
SKY7422	a/α	MSH4(paradoxus)-FLAG-HpHMX4	This study
SKY7424	a/α	MSH4(arboricola)-FLAG-HpHMX4	This study
SKY7425	a/α	MSH4(bayanus)-FLAG-HpHMX4	This study
SKY7481	a/α	msh4Δ::HpHMX4	This study
SKY7445	a/α	THR1/THR1::m-Cerulean-TRP1 CEN8/CEN8::tdTomato-LEU2 ARG4/ARG4::GFP*-URA3	This study
SKY7446	a/α	MSH4(SK1)-FLAG-HpHMX4 THR1/THR1::m-Cerulean-TRP1 CEN8/CEN8::tdTomato-LEU2 ARG4/ARG4::GFP*-URA3	This study
SKY7447	a/α	MSH4(paradoxus)-FLAG-HpHMX4 THR1/THR1::m-Cerulean-TRP1 CEN8/CEN8::tdTomato-LEU2 ARG4/ARG4::GFP*-URA3	This study
SKY7450	a/α	MSH4(arboricola)-FLAG-HpHMX4 THR1/THR1::m-Cerulean-TRP1 CEN8/CEN8::tdTomato-LEU2 ARG4/ARG4::GFP*-URA3	This study
SKY7451	a/α	MSH4(bayanus)-FLAG-HpHMX4 THR1/THR1::m-Cerulean-TRP1 CEN8/CEN8::tdTomato-LEU2 ARG4/ARG4::GFP*-URA3	This study
SKY7452	a/α	msh4Δ::HpHMX4 THR1/THR1::m-Cerulean-TRP1 CEN8/CEN8::tdTomato-LEU2 ARG4/ARG4::GFP*-URA3	This study

SK1 background ho::LYS2, lys2, ura3, leu2, Trp::hisG
